# A thermally insulating vermiculite nanosheet–epoxy nanocomposite paint as a fire-resistant wood coating†

**DOI:** 10.1039/d1na00207d

**Published:** 2021-06-07

**Authors:** Abimannan Sethurajaperumal, Anagha Manohar, Arghya Banerjee, Eswaraiah Varrla, Hao Wang, Kostya (Ken) Ostrikov

**Affiliations:** Nanosheets and Nanocomposites Laboratory, Department of Physics and Nanotechnology, SRM Institute of Science and Technology Kattankulathur, Chengalpattu Tamil Nadu 603203 India eswarail@srmist.edu.in; Centre for Future Materials, University of Southern Queensland Toowoomba QLD 4350 Australia; School of Chemistry and Physics, QUT Centre for Materials Science, Queensland University of Technology (QUT) Brisbane QLD 4000 Australia kostya.ostrikov@qut.edu.au

## Abstract

Conventional fire-retardant composite coatings are typically made of organic-based materials that reduce flame spread rates. However, the associated chemical reactions and starting precursors produce toxic and hazardous gases, affecting the environment and contributing to climate change. Wood is one of the most common materials used in construction and households, and thin-film fire-retardant coatings are needed to protect it from fire. Here, we derive high-performance nanocomposite paint-based coatings from naturally occurring and highly insulating layered vermiculite. The coatings are made using different weight percentages of shear-exfoliated vermiculite nanosheets in an epoxy matrix and are brush-coated onto teak wood. A series of tests using coated wooden rods and standard fire retardancy tests confirm a reduction in flame spread and combustion velocity with minimal toxic smoke release. Samples coated with the vermiculite/epoxy nanocomposite paint resist fire propagation, and post-combustion analysis indicates their resistance to thermal degradation. Our results offer a novel and eco-efficient solution to minimize the flame propagation rate, enhancing char development, and expand the scope of applications of ultra-thin vermiculite in nanocomposite coatings as a fire retardant, exploiting its low thermal conductivity in thermal insulation systems.

## Abstract 2

A nanocomposite made of exfoliated vermiculite nanosheets and epoxy is developed for extinguishing fire and protecting wood from fire degradation.

## Graphical abstract

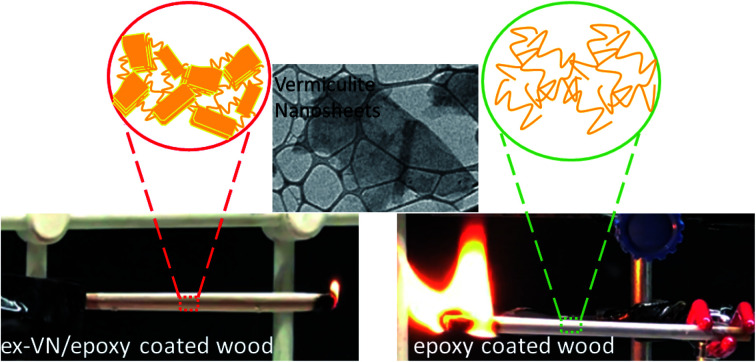

## Introduction

Since the discovery of graphene, a variety of 2D nanomaterials with interesting properties have been continuously evolving. In fact, their properties are controlled by the number of layers and the functional groups embedded in them.^1–3^ The exfoliation of layered nanosheets with unique thermal and electrical properties for diverse target applications is rapidly gaining momentum.^4^ Some of these layered materials are mono-elemental or bi-elemental, while others have complex structures and contain many elements.^5^ One such example is the phyllosilicate family. Phyllosilicates are the group of minerals that form parallel sheets of silicate tetrahedra, *e.g.* talc, mica, serpentine and other clay minerals.^6^ Vermiculite is an exciting and naturally occurring clay-based layered material.^7^ It has incredibly low thermal conductivity due to phonon scattering at ordered interfaces as well as a very low density.^8^ Recently, Paolo *et al.*^9^ reported the thermal insulation properties of vermiculite nanosheets exfoliated in acidic solvent from their 3D precursors. They found that the exfoliated vermiculite nanosheets (<0.1 W m^−1^ K^−1^) had ultra-low thermal conductivity and proposed possible applications in thermal insulation and protection. Therefore, vermiculite nanosheets can be used effectively as nanofillers in epoxy or polymer matrices.^10,11^

Wood is used in construction and interior design due to its unique characteristics such as natural insulation and low-energy production; however, it is highly flammable.^12^ Phosphorus-based coatings that form char when ignited and dramatically swell when exposed to fire are widely used in passive fire protection of structures, but at the cost of releasing toxic gases.^13^ Layered double hydroxide nanofillers have been extensively studied for fire retardant applications owing to the ability of the divalent and trivalent cations to suppress flame.^14,15^ Carbon nanotube (CNT) and graphene-based polymer composites are good fire retardants that use the barrier mechanism to retard flame spread.^16,17^ Carbon nanotubes are expensive to synthesize. Experimental difficulties in aligning high-thermal-conductivity graphene nanosheets parallel to the substrate surface and volatility in reducing heat resistance limit their use in thermal protection systems.^18^

Exfoliated boron nitride (BN) nanosheets,^19^ aerogels,^20^ polyurethane-based vermiculite composites,^21^ heterostructures^22^ and clay along with polyethylenimine and chitosan^23,24^ have been effectively used in thermal insulation and flame retardant applications to reduce thermal resistance, and the addition of vermiculite has been found to delay the decomposition temperature and impede oxygen supply. To the best of our knowledge, there is presently no coating material that satisfactorily protects wood against fire using a sustainable two-dimensional material with ultra-low thermal conductivity. The range of sustainable materials and methods to develop next-generation fire-resistant materials is continuously expanding.^25^

In this work, high-aspect-ratio exfoliated vermiculite nanosheets (ex-VN) are prepared by shear exfoliation of the bulk precursor in an aqueous medium, and a vermiculite-based nanocomposite paint is formulated using epoxy as the matrix. Due to its natural abundance, low cost and ultra-low thermal conductivity, the fire-retardant properties of wood are enhanced after coating with the ex-VN/epoxy nanocomposite paint. The addition of ex-VN to the epoxy leads to flame-extinguishing properties, reduced smoke production in the combustion test, and protection of the underlying structure.

## Results and discussion

### Vermiculite nanosheets and nanocomposite coating

**Fig. 1:**
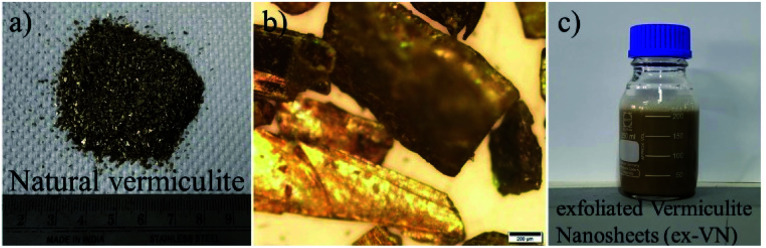
The shear exfoliation of the naturally occurring phyllosilicate mineral. (a) A photograph of the as-received bulk vermiculite sample (grade 5), (b) an optical microscope image of vermiculite, showing its sharp edges and shiny surface (scale bar: 200 μm), and (c) an aqueous dispersion of exfoliated vermiculite nanosheets in a water–surfactant system.

The as-received bulk vermiculite had a light brownish-yellow appearance, as shown in Fig. 1a. The optical microscope image shows a shiny surface due to the presence of metallic ions and large crystals (Fig. 1b). The layers in vermiculite can be separated using shear/sonic energy in solvents. Shear exfoliation is known to delaminate layered materials into thin nanosheets.^26^

To avoid the aggregation or sedimentation of the exfoliated vermiculite, we used a surfactant to stabilize the vermiculite nanosheets in water; a photograph of the dispersion is shown in Fig. 1c. Here, the role of the anionic surfactant is to overcome the attractive van der Waals forces between the exfoliated nanosheets, which are stabilized due to electrostatic repulsive forces.^27^ To analyze the quality of the nanosheets and lateral dimensions of the exfoliated vermiculite, TEM was performed.

**Fig. 2:**
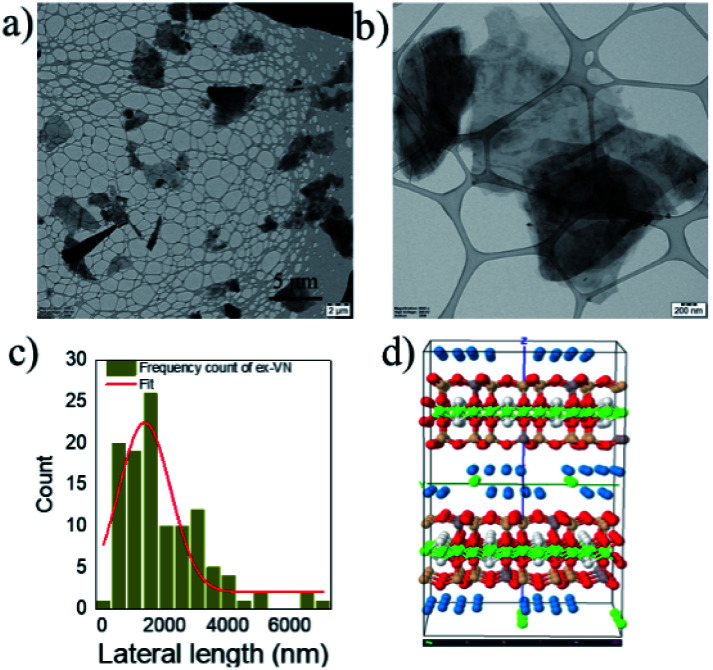
The unique structure of vermiculite, ideal for epoxy nanocomposites. (a and b) Low- and high-magnification TEM images of the exfoliated vermiculite nanosheets, (c) a histogram of the number of exfoliated vermiculite nanosheets as a function of the lateral nanosheet length, and (d) the structure of vermiculite, showing the sandwiched networks of tetrahedral and octahedral sheets (image adapted from https://virtual-museum.soils.wisc.edu/display/vermiculite).

As shown in the images in Fig. 2a and b, the exfoliated nanosheets are thin and transparent. The average lateral length is an essential factor in nanofiller-based epoxy composite applications. We measured the lateral length of ∼100 vermiculite nanosheets from TEM images using ImageJ software. The statistical distribution shown in Fig. 2c indicates that ∼70% of the nanosheets have lengths of over one micron, with an average lateral length of 1350 nm ± 149 nm. The structure of vermiculite is identical to that of mica and talc with a 2 : 1 composition of magnesium-based octahedral sheets sandwiched between two tetrahedral sheets of silicon,^28^ as shown in Fig. 2d. It has a layered structure and weakly bonded exchangeable cations, and water molecules are present between these layers. The results demonstrate that the bulk vermiculite samples were successfully exfoliated in the water–surfactant solution using a simple shear exfoliation process.

**Fig. 3:**
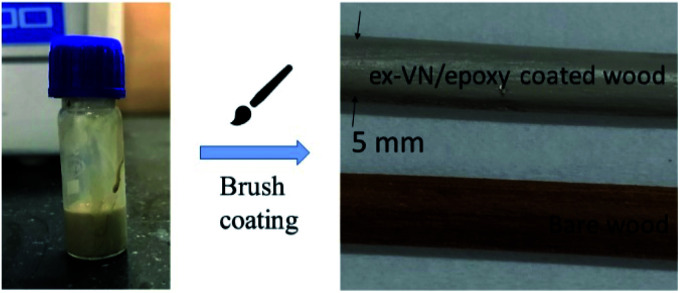
A schematic diagram of brush-coating the epoxy nanocomposite filled with the exfoliated vermiculite nanosheet ink over wood.

Subsequently, a solvent-exchange procedure was carried out to exchange the solvent from water to acetone. Fig. 3 shows the ex-VN/epoxy paint. The paint was prepared by first dispersing the vermiculite nanosheets in the epoxy, followed by bath sonication for 30 min to achieve homogenization. Hardener was then added to the ex-VN/epoxy to make the paint, and the mixture was again sonicated for 30 min. This composite paint suspension was coated directly on the wood with a fine brush. To study the fire-retardant properties of the ex-VN/epoxy nanocomposite, experiments were performed using two sets of coatings: an epoxy-only coating (control sample), and a series of coatings containing varying weight percentages of ex-VN (5%, 10%, 20%, and 30 wt%). To test the effect of the coatings on fire resistance properties^29^ such as thermal degradation^30^ and combustion velocity, fire tests were performed on coated wooden rods, including rods coated with the control sample. Brush-coating using brush strokes in a single direction yielded uniform coatings, as can be seen from the surface smoothness of the coated rod.

**Fig. 4:**
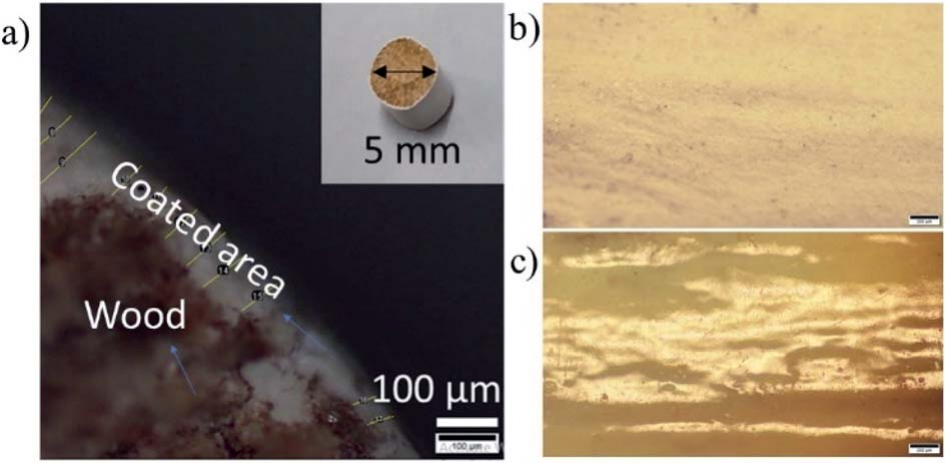
Optical microscopy studies of the nanocomposite paint coating. (a) A cross-sectional microscopic image of an ex-VN/epoxy-coated wooden rod; the inset shows a photograph of the sample. (b) The smooth surface of the ex-VN/epoxy sample and (c) the rough surface of the control sample with the epoxy and cross-linking agent (b and c, scale bar: 200 μm).

The thickness of the coated film was measured using an optical microscope for samples on which 5, 7 and 15 coatings of the 5 wt% ex-VN/epoxy had been brush-painted. A representative optical microscopy image is shown in Fig. 4a. The thickness was computed from the images by measuring the thickness at 30 different points, and the average values were ∼85 μm for the sample with 5 coatings and ∼104 μm for the sample with 15 coatings. In the case of the 30 wt% samples, most of the paint is composed of the epoxy matrix, making the coating smooth and dense; the top view of the surface in Fig. 4b confirms the smoothness of the paint coating. In contrast, the surface of the control sample was rough, as shown in Fig. 4c. Thinner paints generally have a greater tendency to flow on the coated surface, aided by gravity. Therefore, when the samples are rested for curing, lumps occur at specific points on the rod surface. The brush-coated samples also exhibit a few inconsistencies since the samples were coated by hand and the wood surface was polished using sandpaper, thereby resulting in a somewhat non-uniform thickness due to the varying force applied *via* brush strokes on the wooden surface.

### Standard fire testing and thermal degradation

**Fig. 5:**
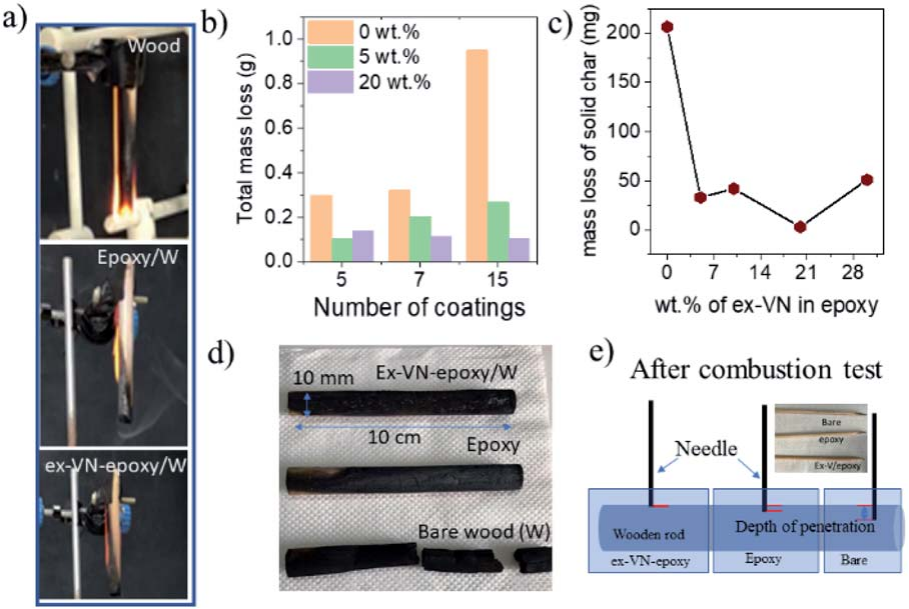
The reduction in thermal degradation using the exfoliated vermiculite nanosheets–epoxy nanocomposites. (a) Photographs of samples after ignition in the standard UL-94 test: bare wood (top), epoxy-coated wood (middle), and ex-VN/epoxy-coated wood (bottom). (b) The total mass loss of the wooden rod sample after the combustion test *vs.* the number of coatings. (c) The mass loss of solid char from wooden rods as a function of the weight percentage of ex-VN in the epoxy. (d) Photographs of the coated wood samples after the ignition test. (e) A schematic diagram of the penetration of a sharp pin into the wood samples after the combustion test (the inset shows real images of pins inserted to different depths).

The Underwriters Laboratory-94 (UL-94) test is a standard plastics flammability test based on the tendency of material to extinguish flame and resist dripping.^31,32^ It also provides the time taken for the flame to spread.^33^Fig. 5a shows bare wood, control sample coated wood and ex-VN-epoxy coated wood. A schematic of the UL-94 experimental set up is shown in the ESI (Fig. S2†). The sample is exposed to flame for 10 s, and the time required for the flame on the sample to be extinguished is recorded as *t*_1_. After the flame is completely extinguished, the same process is repeated, and time *t*_2_ is noted. Often, even after the flame is extinguished, a reddish afterglow remains, which slowly fades away. The time taken for this glow to disappear is recorded as time *t*_3_. Depending upon the values of *t*_1_, *t*_2_, and *t*_3_ obtained from the UL-94 test, a polymer composite can be given a fire retardant rating of *V*_0_, *V*_1_, or *V*_2_. Materials that exhibit slow burning or extinguish themselves suddenly without any dripping are given the highest UL-94 ranking.^34,35^

Rod-shaped wooden samples with a length of 10 cm and diameter of 10 mm were rigidly fixed to the stand. In the UL-94 test, if the flame is extinguished in less than 10 s after the first ignition and less than 50 s after the second ignition, then it is ranked as *V*_0_ category, which is considered to be the best class of fire-retardant materials.^36^ The sample coated five times with the 20 wt% ex-VN-filled epoxy composite was able to extinguish the flame within 10 s upon the first exposure to flame in the UL-94 test, qualifying it as *V*_0_.

The control sample did not display any fire extinguishing properties and had to be forcefully extinguished after the entire length of the rod was burnt 67 s after the first ignition. On the other hand, the ex-VN/epoxy-coated wood sample extinguished the flame in just 10 s, while the bare wood sample extinguished the flame in 31 s. On the vermiculite nanosheets–epoxy-coated sample, the flame propagated over only 4% of the wooden rod after the first ignition. This more favourable time duration shows that the ex-VN/epoxy nanocomposite coating can hold the fire static for a longer time by forming a physical barrier to propagation.^37^ Since combustible and non-combustible gases were also released, the total and solid mass loss of the sample were quantified by tapping the samples with minimal force to see how much black char disintegrated from the edges of the tested samples. The results are shown in Fig. 5b and c. It was noted that there was a sharp decrease (∼98%) in the amount of char formed during the combustion of the ex-VN/epoxy coated samples compared to that for the epoxy-coated control samples. The control samples show a marked 68.5% increase in thermal degradation as the number of coatings increases from 5 to 15. This increase is due to the higher amount of epoxy, which is polymeric and flammable, on the substrate. In the case of the 5 wt% samples, with an increasing number of coatings, the thermal degradation is increased as well. For this lower concentration of ex-VN, a smaller number of coatings works better. The trend is different when the concentration of ex-VN is high. In the case of 20 wt% ex-VN, it was observed that the thermal degradation decreased by 24% when the number of coatings was increased from 5 to 15. This implies that the higher amount of ex-VN in the thicker coatings was able to successfully counteract the heat produced by the pyrolysis of epoxy matrix. When a higher percentage of ex-VN is used in the epoxy, larger amounts of water molecules are present, which undergo an endothermic dehydration process to remove some of the heat generated during the combustion process.

As shown in Fig. 5d, the epoxy control samples developed visible cracks along the length of the wood, and their structure deteriorated, indicating that the coating was not able to prevent the flame from penetrating into the wood structure and inducing substantial degradation. When the ex-VN/epoxy-coated wood samples were tapped after the tests, shedding of the loosely adhered black-coloured char from their surface occurred. Although the surface of the ex-VN/epoxy coated wood turned black after the fire test, the core of the wood remained intact, and cracks did not appear, in stark contrast to the other samples. No dripping was observed for the ex-VN/epoxy coated samples.

We performed a simple experiment to determine the depth of the structure disintegration. Sharp wooden pins were inserted at various points on the surface of the tested samples, as shown in Fig. 5e, and the depth to which the pin penetrated was measured. When the bare wood sample was pierced, it was unable to withstand the minimum pressure when the pin was inserted and broke into pieces. This experiment suggests that the pin was able to travel through the entire diameter of the rod, *i.e.*, 10 mm. In the case of the epoxy-coated samples, the pin at the centre point travelled a distance of >1 mm at the edges that were exposed to the flame. The same test was also performed on the ex-VN–epoxy coated samples. It was observed that at all points of the sample, the pin travelled <1 mm. This test leads to the conclusion that the vermiculite nanosheets are able to successfully reduce the penetration of the flame into the wood sample.

### Flame extinguishing time and flame-spread characteristics

**Fig. 6:**
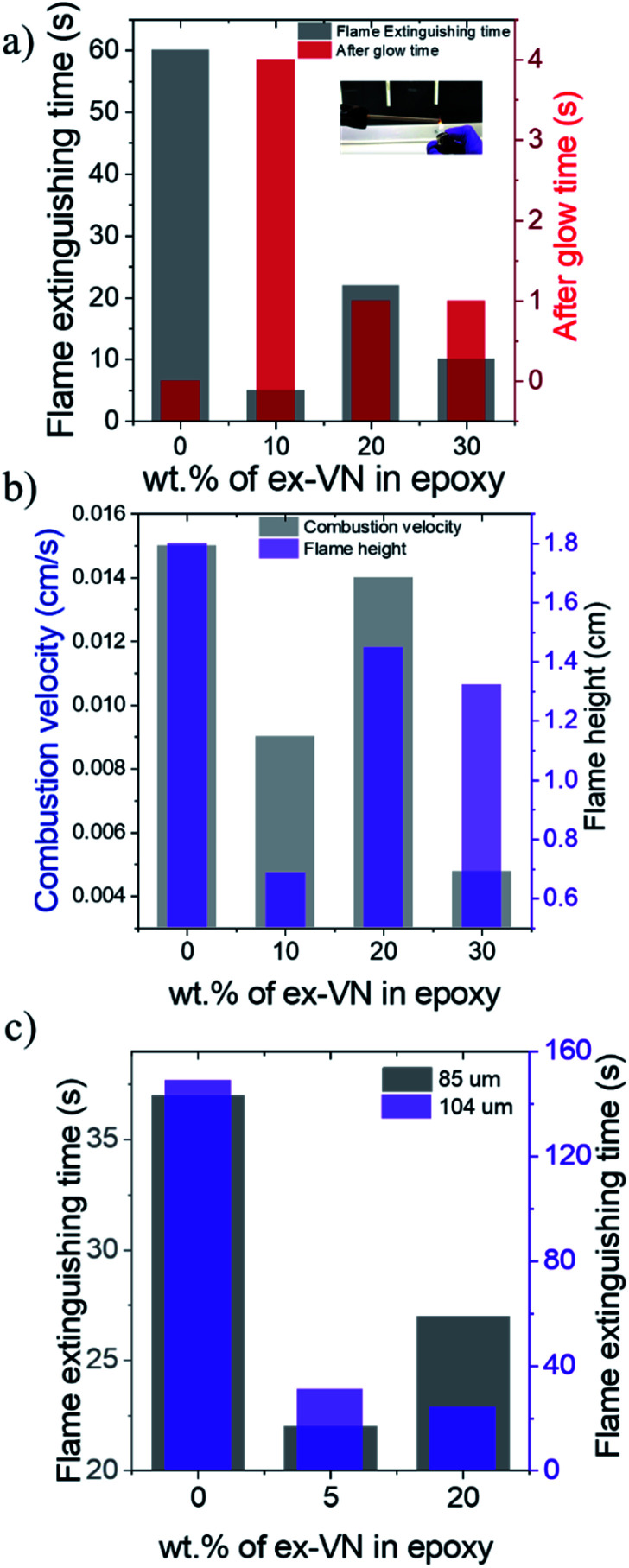
The fire-retardant properties of ex-VN–epoxy nanocomposites. (a) The fire extinguishing times and after-glow times of 5 mm wooden rods brush-coated five times with epoxy composite coatings filled with different wt% levels of ex-VN (the inset shows a photograph of the experimental set up). (b) Combustion velocity and flame height values of composite-coated samples *vs.* vermiculite nanosheet wt%. (c) Fire extinguishing time for 5 mm wooden rods with ∼85 μm and ∼104 μm thick ex-VN–epoxy coatings after being exposed to a flame for 6 s.

Fire tests were conducted on the epoxy and ex-VN/epoxy nanocomposites with different weight percentages of the nanofiller to measure the flame extinguishing time, combustion velocity and flame height. The set-up used to measure the fire-resistance properties is shown in the inset of Fig. 6a. The epoxy-coated sample showed no extinguishing properties and had to be extinguished forcefully 60 s into the experiment. Photographs of the samples for the flame height measurements are shown in Fig. S5.† The sample with the optimum weight percentage of exfoliated vermiculite nanosheets in epoxy showed the highest resistance towards catching fire when ignited for the first time. This response is due to the good coverage of the vermiculite nanosheets embedded in the epoxy and the reduction of thermal conductivity to the ultra-low regime upon exfoliation. The 10 wt% sample took 5 s to self-extinguish, while the 20 wt% and 30 wt% samples took 22 s and 10 s to self-extinguish, respectively, as shown in Fig. 6a. The observed variation in the self-extinguishing time at higher weight percentages of ex-VN is due to the compromise in the formation of a compact char layer on the wooden surface. The attractive van der Waals forces between the vermiculite nanosheets cause aggregation at higher weight percentages. This leads to larger tortuous paths at the optimum loading and shorter paths at higher loading (Fig. S9†). At higher weight percentages, this aggregation of the layered material affects the barrier formed by char, thereby creating pathways through which heat can travel and sustain the pyrolysis reaction. Also, the dispersion of higher loadings of nanosheets in epoxy could increase the viscosity resulting in inhomogeneous film formation at higher weight percentages, leading to variation in the self-extinguishing time. Interestingly, the afterglow time for the ex-VN filled epoxy nanocomposite samples was much longer than that of the epoxy control sample. This result indicates that not only does the film coating resist fire, but also holds the structure intact for a longer period without disintegrating. During the combustion of wood with phosphorus-based coatings, the smoke density is high and toxic chemicals are released.^38^ As shown in Fig. 6b, the epoxy control sample shows a high combustion velocity of 0.015 cm s^−1^, which is reduced by 40% in the 10 wt% sample and by 68.3% in the 30 wt% sample. There is a reduction in the spread of flame with increasing vermiculite nanosheet content in the epoxy, which gives rise to a mechanism in which the heat is dissipated gradually during the combustion process. Fig. 6b shows the flame test results, revealing that there is a ∼62% decrease in the flame height for the 10 wt% samples, a ∼20% decrease for the ∼20 wt% samples, and a ∼27% decrease for the 30 wt% samples.

A substantial reduction in smoke release and other harmful chemical fumes is observed. This reduction in smoke can be attributed to the inorganic nature of the vermiculite nanofiller. Its water content and intercalated ions enhance the formation of the char layer post-combustion. In the absence of the coating, the char layer formed is quite weak and often cracks into powdery ash, allowing gases to be released from the wood surface and form smoke. However, the presence of the coating leads to the formation of an enhanced thick char layer, which acts as an insulation barrier, preventing the gases from escaping and mixing with the air to form smoke.^8,39^ As shown in Fig. 6c, as the thickness of the coating was increased, the fire extinguishing time shows a decreasing trend with increasing wt% of vermiculite nanosheets, reflecting the fire-retarding behaviour. Comparing the ∼104 μm thick ex-VN/epoxy nanocomposite films to the control sample, we see a ∼79% decrease for the 5% sample and an ∼83% decrease for the ∼20% sample.

Wooden rods of varying diameter were coated five times using the 10 wt%, 20 wt%, or 30 wt% ex-VN–epoxy nanocomposite. The optimum fire-retardant effect in terms of combustion velocity and flame spread was observed for the 5 mm diameter rods. The thin 3 mm rod samples have the advantage of having less fuel for combustion. The 30 wt% nanocomposite coating shows superior fire-retardant properties, with the flame self-extinguishing within 2 s when ignited in the case of the 3 mm wooden rods, while the 5 mm samples took 8 s longer to extinguish. The flame spread is faster on the 3 mm wood samples, indicating a high combustion velocity. Hence, the 5 mm wooden rods can hold the flame static for a longer time and prevent it from spreading, as the cross-section of the sample is larger. As a result, the flame spreads gradually on the samples with larger cross-sections for the same coating thickness.

**Fig. 7:**
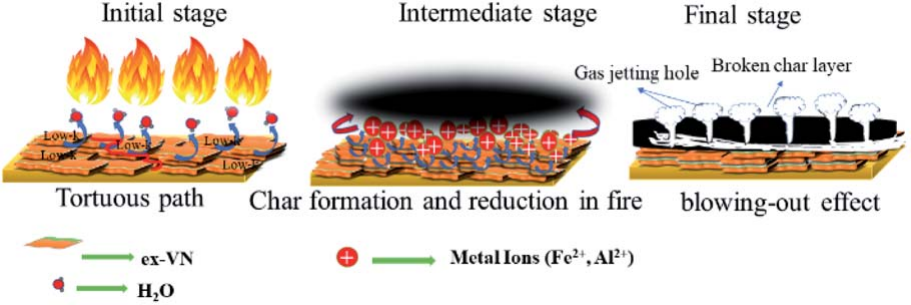
A plausible fire-retardant mechanism using exfoliated vermiculite nanosheets in epoxy.

The reason that the exfoliated vermiculite nanosheets work as a fire-retardant film coating on wooden surfaces is a combination of three important factors. The mechanism is schematically depicted in Fig. 7. Firstly, vermiculite exhibits exceptionally low thermal conductivity compared to most inorganic layered materials. The high aspect ratio and low thermal conductivity (*k*) values of exfoliated vermiculite allow it to act as a physical barrier forming interconnections over the wood surface.^9^ The dispersion of the compact ex-VN network is shown in Fig. S10.† FTIR spectra suggest that weak interactions and grafting exist between the filler and the epoxy (Fig. S11†). This would force flammable gases/products to take a tortuous path to react with the surface beneath.^37^ In addition to thermal conductivity, thermal diffusivity and thermal effusivity are important properties for heat transmission through the material and its surroundings. Thermal diffusivity through a material is expressed as:
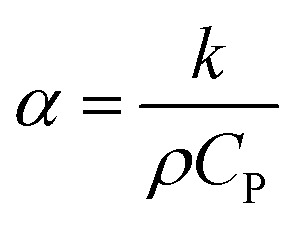
where *α* is the thermal diffusivity (m^2^ s^−1^), *k* is the thermal conductivity of the nanofiller (∼0.1 W m^−1^ K^−1^),^9^*ρ* is the density of the nanofiller (225 kg m^−3^) and *C*_P_ is the specific heat capacity of the nanofiller (∼108 J kg^−1^ K^−1^). By substituting these values into the equation, the thermal diffusivity of vermiculite is calculated to be ∼4.11 × 10^−6^ m^2^ s^−1^. This diffusivity value is much lower than the reported values.^40^ During combustion, an ultra-small amount of thermal energy is transmitted through the material. Thermal effusivity is defined as:
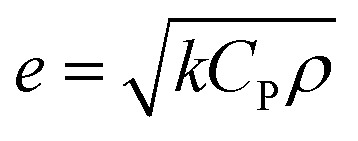
and its value is 1558 W (s)^1/2^ m^−2^ K^−1^. Although this value is slightly higher than the reported values,^40^ in terms of thermal conduction and mass transfer, the low-regime thermal conductivity leads to enhanced fire-retardant properties.^41^

Heat flux is an important factor in the combustion analysis of materials, particularly wood-like structures, and is best described by the fire triangle (fuel, oxygen and heat). It is understood as the flow of energy per unit area per unit time (kW m^−2^). The temperature of a lighter flame is different in different areas of the flame. The high temperature zone of the lighter flame, *i.e.*, the non-luminous zone, was used for the combustion analysis of wood. The temperature of the non-luminous zone is above ∼1500 K. We found that most of the lighter heat flux values in the literature were greater than ∼50 kW m^−2^.^42^ Thus, we used 50 kW m^−2^ as the approximate heat flux value to study the fire-retardant property of the wooden rods. Additionally, most materials are ignited with a heat flux between 10 and 20 kW m^−2^.^43^ In cone calorimetry studies, heat flux values in the range of 10–100 kW m^−2^ are typically used to study the peak heat release rate (PHRR), mass loss rate and ignition time of FR materials. Our observations of reductions in the mass loss, flame height, combustion velocity and smoke density corroborate the cone calorimetry measurements. These parameters were effectively used to evaluate the fire-retardant properties of vermiculite nanosheets–epoxy-coated wood.

Here, we used the one-dimensional (1D) steady-state equation to probe the heat flux values through the vermiculite–epoxy-coated wooden rod. We considered the 1D steady-state because the rod dimensions seems to be 1D-like in nature.

**Fig. 8:**
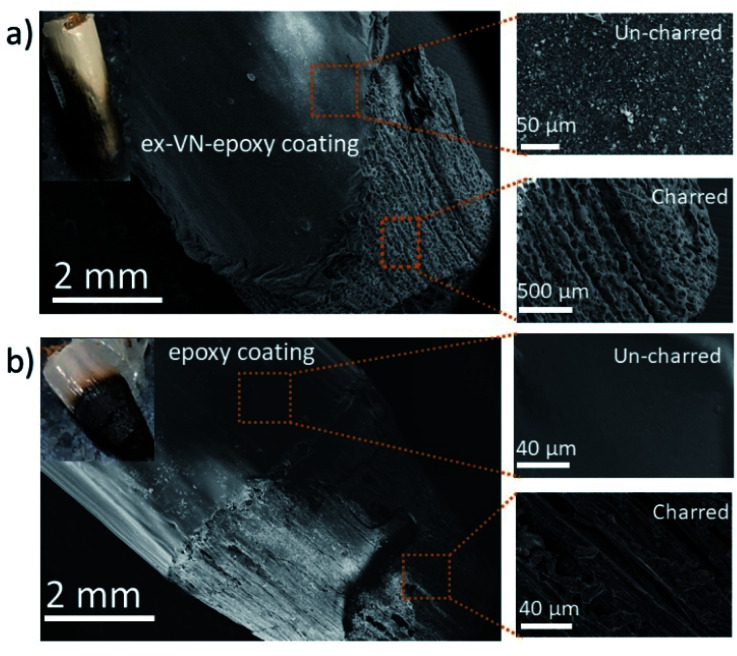
Characterization of char after the combustion tests. Scanning electron microscopy images of (a) a 20 wt% ex-VN–epoxy-coated wooden rod and magnified images depicting its morphology in charred and un-charred regions and (b) control epoxy-coated wood.

The heat flux formula for 1D conduction is:
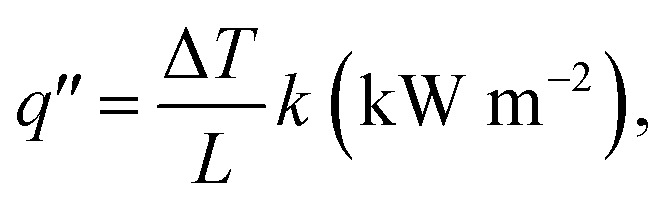
where Δ*T* is the temperature difference (*T*_2_ − *T*_1_), *T*_2_ is the temperature of the wood on the ignited side, and *T*_1_ is the temperature of the other side of the wooden rod. Here, *T*_2_ is ∼1000 K (close to the lighter temperature) and *T*_1_ is ∼300 K (room temperature). *L* is the length of the wooden rod (here, the length up to the point of char formation was measured, *i.e.*, ∼1 cm), and *k* is the thermal conductivity of ex-VN–epoxy composites (∼0.1 W m^−1^ K^−1^). After substituting these values, the steady-state heat flux through the wooden rod was found to be ∼7 kW m^−2^, which is lower than that of most of the nanomaterials used for the study. Secondly, the presence of water molecules, which make up ∼10% of the composition between the layers of vermiculite, helps to reduce the release of toxic gases by reacting with them to produce less harmful by-products; it also reduces the heat release by absorbing heat for evaporation.^8^ Thirdly, under the conditions in which the ex-VN/epoxy nanocomposite coating catches fire, char formation occurs. The pyrolysis chain reaction is not sustained, thereby reducing thermal degradation to a great extent. During the combustion, vermiculite layer creates more char due to the metal ions (Mg^2+^, Al^2+^) in its structure.^44,45^ The produced char forms a protective layer on the substrate, which cuts off the oxygen supply, thus inhibiting the combustion reaction and extinguishing the flame. The black char between the ignition source and the material that has not undergone combustion acts as an oxygen-trapping barrier.^46,47^ As a result, the trapped oxygen is unable to reach the wooden surface beneath, and the combustion process does not take place in the absence of oxygen. It has also been noted that the ability of vermiculite to retain its structure is maintained even after repeated ignitions. To probe the fire-retardant mechanism, the surfaces of a wooden rod coated with ex-VN and a control sample were analyzed using electron microscopy, and the images are shown in Fig. 8a and b. The magnified regions clearly show that the char on the coated wood region has a distinct morphology compared to that of the control sample. After the combustion test, the uncharred region of the epoxy-controlled sample has a smooth morphology, whereas the ex-VN/epoxy-coated sample has a rough surface due to the random distribution of vermiculite nanosheets.

**Fig. 9:**
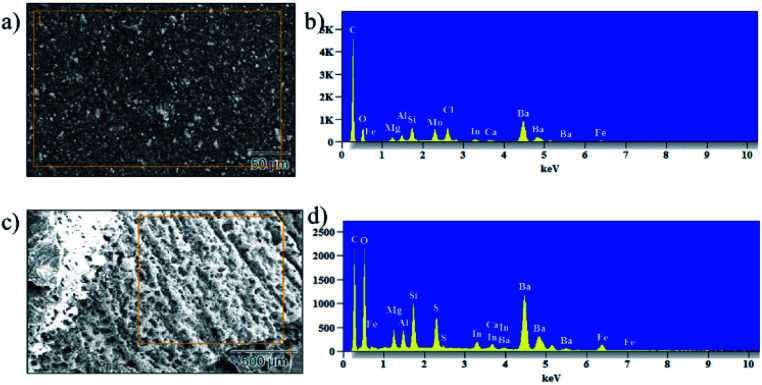
The morphology and elemental composition of 20 wt% ex-VN/epoxy-coated wood before (a and b) and after (c and d) combustion tests.

Vermiculite has a variety of metal ions and few interchangeable cations in its structure. To confirm the presence of nanosheets in the epoxy, we conducted energy dispersive X-ray analysis (EDAX) and elemental mapping; the resulting images are shown in Fig. 9a, b and S3† shows elemental mapping of the un-charred and charred regions of the control and ex-VN/epoxy-coated samples. These results confirm the presence of Si, Al, Mg, and Ti metals, which are part of the vermiculite structure, and other interchangeable cations like Ca and K. We carried out elemental mapping on the charred portion. We knew that the charred portion was formed by lightweight carbon residue. Interestingly, we found the elements of vermiculite in the charred region in addition to carbon, and an increased concentration of element oxygen, which indicates the presence of a compact layer of char in which metal is oxidized.

The inclusion of the exfoliated vermiculite nanosheets in the epoxy matrix successfully introduces flame-retardant characteristics to the nanocomposite, such as decreased combustion velocity, flame extinguishing, decreased flame height and heat release rate, and suppression of toxic smoke release, as well as reduced thermal degradation of the wooden sample. Demonstration of the fire-retardant behaviour of wood with and without ex-VN in epoxy is shown in the ESI Video.† One of the most important characteristics of a fire-retardant material is its ability to self-extinguish flame. As depicted in Fig. 9 and from our observations during fire testing of the ex-VN/epoxy composite, the fire-retardant material releases non-flammable gas *via* a broken char layer, which is known as the blowing-out effect.^48^ Vermiculite contains fire-resistant compounds such as SiO_2_, Al_2_O_3_ and MgO, which are responsible for the formation of inert gas from the char layer. During combustion, vermiculite releases H_2_O, CO_2_, NO and SO_2_.^49^ These gases are all well-known fire extinguishers.

The jetting of these gases outward from the char layer, *i.e.*, the condensed portion, extinguishes the fire.^50^ During combustion, a thermally stable thin layer is formed between the wood surface and the fire. This char layer insulates the wood from fire and instantaneously forms non-flammable gases inside the char. The accumulation of the non-flammable gases in char leads to increased pressure, and these gases then completely extinguish the fire.^49^ In our experiments, there was a remarkable decrease in the thermal degradation of the wooden substrate beneath the coating due to the introduction of vermiculite nanosheets in the nanocomposite. The heat flow always chooses the path which offers less resistance. Vermiculite provides more resistance to heat flow through the coating due to its low value of through-plane thermal conductivity. For this reason, heat does not travel across the surface of the coating. Therefore, the coating can prevent the heat from penetrating into the wooden substrate, thus preserving its integrity after the fire testing. Thus, vermiculite can stop heat from reaching deeper into the samples by forming a physical barrier to heat flow. This is indicative of the fact that vermiculite can withstand heat without undergoing any considerable chemical changes or damages to its internal structure.

## Experimental section

### Materials

Bulk vermiculite samples were received from Tamil Nadu Minerals Limited, Chennai, India. Apcolite 2 pack epoxy finish was purchased from Asian Paints. Sodium dodecyl benzene sulphonate (SDBS) was purchased from Sigma Aldrich. Acetone with 99.5% purity was used in this work and was purchased from S. R. L. Chemicals. Distilled water was used throughout the experiments. Wood samples with a length of 10 cm and diameters of 3 mm, 5 mm and 10 mm were procured from local woodworks, and commercial emery paper was used to polish their surfaces before painting. A commercial lighter was used for fire testing.

### Preparation of exfoliated vermiculite nanosheets (ex-VN)

In this work, we used a liquid-phase exfoliation technique, namely, shear exfoliation, to delaminate the bulk vermiculite into thin layers of the parent component in a water/SDBS surfactant dispersion.^26^ For this, initially, 10 g of grade 5 (fine particle, ∼1 mm) bulk vermiculite was added to 500 ml of water with 0.8 mg ml^−1^ dissolved SDBS. This dispersion was subjected to exfoliation using a kitchen blender for 1 h with an on and off timer to avoid heating effects. After this step, the resultant dispersion was allowed to stand overnight, and the supernatant was recovered using a Pasteur pipette for further dispersion analysis and characterization; the sediment was discarded. Centrifugation of the resultant dispersion was performed at 6000 rpm for 30 min using a Thermo Scientific Sorvall ST8R small benchtop centrifuge to recover the exfoliated vermiculite nanosheets in paste form; the nanosheets were then dried in a vacuum oven at 60 °C for ∼8 h. The exfoliated vermiculite nanosheets were redispersed in acetone using bath sonication. This process was repeated twice to transfer the material from the aqueous solution to acetone solvent, and bath sonication was applied for 30 min to homogenize the sample.

### Preparation of ex-VN/epoxy nanocomposites

The coating paint was formulated using a commercial epoxy (Asian Paints) filled with various weight percentages of ex-VN. The coating was prepared *via* the *in situ* polymerization of the epoxy monomer with a hardener in the presence of the nanofiller. Acetone is used as a paint thinner and solvent medium for ex-VN. To prepare the epoxy/ex-VN nanocomposite, 50 mg of ex-VN (for 5 wt%, the total weight of the composite was 1 g) was initially added to 634 mg of epoxy and bath-sonicated (Branson, 40 kHz, 70 W) for 30 min to disperse the nanofiller in the epoxy. After this step, 316 mg of hardener (3 : 1 ratio of epoxy to hardener) was added to the ex-VN/epoxy monomer, and bath sonication was applied again to disperse the hardener throughout the system. This process was repeated to prepare the 10, 20 and 30 wt% nanocomposites. Acetone was used in the nanocomposite preparation process for homogenization and for thinning the formulated paint before coating it on the wood. Upon the addition of ex-VN into the epoxy matrix, there was a notable change in the colour of the epoxy. The epoxy–hardener is pure white in appearance, and the ex-VN/epoxy ink is essentially light brown. The mixing of these two results in a change in the colour of the matrix; the coating mix is light brown in colour and becomes russet or dark brown when the weight percentage is high.

### Specimen preparation and brush-coating

All the teak wood samples were treated with electro-coated SiC grain emery paper of 220 grit size to improve the adhesion. The as-received epoxy polymerized with a cross-linking agent was also prepared as per the mixing requirements and used as the control sample. The preparation of the control coating did not require bath sonication as it could be homogenized with mechanical stirring. All the samples were coated using flat brushes. The interval between the application of each coat was approximately 2 min, and the specimens were cured at room temperature for 24 h.

### Characterization techniques

A field emission scanning electron microscope (FE-SEM, Thermo scientific Apreo S) was operated at 15 kV to obtain the morphology, elemental composition, and mapping of the charred and uncharred regions of the coated samples. A turbo-pumped sputter coater (Quorum, Q150T Plus) was used to cover the sample surface with Cr before placing the samples under an electron microscope to avoid charging effects, with a sputtering time of 25 s and a film thickness of ∼7 to 10 nm. Transmission electron microscopy (TEM) analysis of the exfoliated vermiculite nanosheets was carried out using a Hi-Resolution Transmission Electron Microscope (HRTEM, JEOL Japan, Model: JEM-2100 Plus). Images were also obtained using an Olympus BX51 optical microscope. Thermogravimetric analysis (TGA) was carried out using a simultaneous thermogravimetric analyzer (STA 7000, HITACHI) in an Ar atmosphere with a rate of 10° C min^−1^. Fourier transform infrared spectra were obtained using an IRTracer 100 (Shimadzu) in ATR mode. A custom-made Underwriters Laboratory-94 fire retardant standard test was performed on wood samples 10 cm in height and 10 mm in diameter to characterize the fabricated ex-VN/epoxy nanocomposites.

## Conclusions

In this work, we have demonstrated an eco-efficient fire-retardant coating using epoxy filled with shear exfoliated vermiculite nanosheets on a wooden surface. A simple and scalable brush-coating technique was used, and a film thickness of ∼100 μm was achieved. Fire tests were performed on samples with varying weight percentages of vermiculite nanosheet filler (5–30 wt%) in the epoxy matrix to test the effect of the vermiculite loading. The introduction of the low-*k* vermiculite filler into the matrix was found to reduce the flame height and, consequently, the heat released during combustion by up to ∼62% in the case of the 10 wt% sample. The thermal degradation tests showed a decrease of ∼42% due to the addition of exfoliated vermiculite nanosheets. A 68.3% decrease in combustion velocity was observed for the samples with 30% vermiculite by weight in their matrix. It was also observed that the samples without vermiculite showed no self-extinguishing properties and burned to completion, thereby leading to the complete disintegration of the wooden structure beneath the coating. This work opens up new opportunities for using insulating layered 2D materials to resolve common issues related to fire retardants and to develop alternative environmentally friendly, high-performance materials.

## Author contributions

E. V. initiated the idea and designed experiments. A. S. and A. M. prepared vermiculite nanosheets and ex-VN/epoxy composites. A. S. characterized samples by TEM, SEM, EDAX and OM. A. M., A. S. and A. B. performed the fire testing, UL-94 and recorded observations. E. V., A. S., A. M. carried out result analysis and drafted the initial manuscript. E. V., H. W. and K. O. revised the manuscript. All authors contributed to the revision and discussion of the manuscript.

## Conflicts of interest

The authors declare that they have no known competing financial interests or personal relationships that could have appeared to influence the work reported in this paper.

## Supplementary Material

NA-003-D1NA00207D-s001

NA-003-D1NA00207D-s002

## References

[cit1] Li X.-L., Han W.-P., Wu J.-B., Qiao X.-F., Zhang J., Tan P.-H. (2017). Adv. Funct. Mater..

[cit2] Xiong K., Wang P., Yang G., Liu Z., Zhang H., Jin S., Xu X. (2017). Sci. Rep..

[cit3] Ferrari A. C., Bonaccorso F., Fal'ko V., Novoselov K. S., Roche S., Bøggild P., Borini S., Koppens F. H. L., Palermo V., Pugno N., Garrido J. A., Sordan R., Bianco A., Ballerini L., Prato M., Lidorikis E., Kivioja J., Marinelli C., Ryhänen T., Morpurgo A., Coleman J. N., Nicolosi V., Colombo L., Fert A., Garcia-Hernandez M., Bachtold A., Schneider G. F., Guinea F., Dekker C., Barbone M., Sun Z., Galiotis C., Grigorenko A. N., Konstantatos G., Kis A., Katsnelson M., Vandersypen L., Loiseau A., Morandi V., Neumaier D., Treossi E., Pellegrini V., Polini M., Tredicucci A., Williams G. M., Hee Hong B., Ahn J.-H., Min Kim J., Zirath H., van Wees B. J., van der Zant H., Occhipinti L., Di Matteo A., Kinloch I. A., Seyller T., Quesnel E., Feng X., Teo K., Rupesinghe N., Hakonen P., Neil S. R. T., Tannock Q., Löfwander T., Kinaret J. (2015). Nanoscale.

[cit4] Nicolosi V., Chhowalla M., Kanatzidis M. G., Strano M. S., Coleman J. N. (2013). Science.

[cit5] Cygan R. T., Greathouse J. A., Heinz H., Kalinichev A. G. (2009). J. Mater. Chem..

[cit6] Harvey A., Boland J. B., Godwin I., Kelly A. G., Szydłowska B. M., Murtaza G., Thomas A., Lewis D. J., O'Brien P., Coleman J. N. (2017). 2D Mater..

[cit7] Qian Y., Lindsay C. I., Macosko C., Stein A. (2011). ACS Appl. Mater. Interfaces.

[cit8] Suvorov S. A., Skurikhin V. V. (2003). Refract. Ind. Ceram..

[cit9] Janica I., Del Buffa S., Mikołajczak A., Eredia M., Pakulski D., Ciesielski A., Samorì P. (2018). Nanoscale.

[cit10] Balazs A. C., Emrick T., Russell T. P. (2006). Science.

[cit11] Ku H., Wang H., Pattarachaiyakoop N., Trada M. (2011). Composites, Part B.

[cit12] Guo H., Özparpucu M., Windeisen-Holzhauser E., Schlepütz C. M., Quadranti E., Gaan S., Dreimol C., Burgert I. (2020). ACS Sustainable Chem. Eng..

[cit13] Gomez-Mares M., Tugnoli A., Landucci G., Cozzani V. (2012). Ind. Eng. Chem. Res..

[cit14] Gao Y., Wu J., Wang Q., Wilkie C. A., O'Hare D. (2014). J. Mater. Chem. A.

[cit15] Liu S., Fang Z., Yan H., Chevali V. S., Wang H. (2016). Composites, Part A.

[cit16] Fang F., Ran S., Fang Z., Song P., Wang H. (2019). Composites, Part B.

[cit17] Wang Q., Su D. S., Wang D.-Y. (2020). ACS Appl. Nano Mater..

[cit18] Canosa G., Alfieri P. V., Giudice C. A. (2011). Ind. Eng. Chem. Res..

[cit19] Li X., Feng Y., Chen C., Ye Y., Zeng H., Qu H., Liu J., Zhou X., Long S., Xie X. (2018). J. Mater. Chem. A.

[cit20] Chen Y., Qin H., Song J., Liu Z., Liu Y., Pei Q.-X. (2020). Nanoscale.

[cit21] Kirbaş İ. (2020). J. Thermoplast. Compos. Mater..

[cit22] Mohanta M. K., Rawat A., Dimple, Jena N., Ahammed R., De Sarkar A. (2019). Nanoscale.

[cit23] Cain A. A., Plummer M. G. B., Murray S. E., Bolling L., Regev O., Grunlan J. C. (2014). J. Mater. Chem. A.

[cit24] Lazar S., Carosio F., Davesne A.-L., Jimenez M., Bourbigot S., Grunlan J. (2018). ACS Appl. Mater. Interfaces.

[cit25] Puri R. G., Khanna A. S. (2017). J. Coat. Technol. Res..

[cit26] Varrla E., Paton K. R., Backes C., Harvey A., Smith R. J., McCauley J., Coleman J. N. (2014). Nanoscale.

[cit27] Lotya M., King P. J., Khan U., De S., Coleman J. N. (2010). ACS Nano.

[cit28] Valášková M. and Martynkova G. S., Clay minerals in nature-their characterization, modification and application, InTech, 2012, pp. 209–238

[cit29] Lowden L. A., Hull T. R. (2013). Fire Sci. Rev..

[cit30] Gao M., Sun C., Wang C. (2006). J. Therm. Anal. Calorim..

[cit31] Wang Y., Zhang J. (2013). J. Hazard. Mater..

[cit32] Levinṭa N., Vuluga Z., Teodorescu M., Corobea M. C. (2019). SN Appl. Sci..

[cit33] Patel P., Hull T. R., Moffatt C. (2012). Fire Mater..

[cit34] Gao F., Tong L., Fang Z. (2006). Polym. Degrad. Stab..

[cit35] Qiu S., Zou B., Zhang T., Ren X., Yu B., Zhou Y., Kan Y., Hu Y. (2020). Chem. Eng. J..

[cit36] Wang H., Qiao H., Guo J., Sun J., Li H., Zhang S., Gu X. (2020). Composites, Part B.

[cit37] Cao Z.-J., Liao W., Wang S.-X., Zhao H.-B., Wang Y.-Z. (2019). Chem. Eng. J..

[cit38] Liu Q., Wang D., Li Z., Li Z., Peng X., Liu C., Zhang Y., Zheng P. (2020). Materials.

[cit39] Yan L., Xu Z., Zhang J. (2016). Iran. Polym. J..

[cit40] Liu J., Kutty R. G., Zheng Q., Eswariah V., Sreejith S., Liu Z. (2017). Small.

[cit41] Cirpici B. K., Wang Y. C., Rogers B. (2016). Fire Saf. J..

[cit42] Williamson J. W. , Characterizing cigarette lighter flames to reduce unwanted ignition, PhD diss., 2004

[cit43] Gorbett G. E. , Pharr J. L. and Rockwell S., Fire dynamics, Pearson, 2016

[cit44] Wang P.-J., Hu X.-P., Liao D.-J., Wen Y., Hull T. R., Miao F., Zhang Q.-T. (2017). Ind. Eng. Chem. Res..

[cit45] Wang X., Spörer Y., Leuteritz A., Kuehnert I., Wagenknecht U., Heinrich G., Wang D.-Y. (2015). RSC Adv..

[cit46] Jiang S.-D., Bai Z.-M., Tang G., Song L., Stec A. A., Hull T. R., Hu Y., Hu W.-Z. (2014). ACS Appl. Mater. Interfaces.

[cit47] Qian L., Li L., Chen Y., Xu B., Qiu Y. (2019). Composites, Part B.

[cit48] Zhang W., Li X., Yang R. (2012). Polym. Degrad. Stab..

[cit49] Zhang W., Li X., Li L., Yang R. (2012). Polym. Degrad. Stab..

[cit50] Kremenetskaya I., Ivanova L., Chislov M., Zvereva I., Vasilieva T., Marchevskaya V., Semushin V., Slukovskaya M. (2020). Appl. Clay Sci..

