# Supplementary material for: A thermally insulating vermiculite nanosheet–epoxy nanocomposite paint as a fire-resistant wood coating
Source: Nanoscale Adv. 2021 Jun 7;3(14):4235–43. doi: 10.1039/d1na00207d (PMC9417340; doi:10.1039/d1na00207d)
Supplement: NA-003-D1NA00207D-s001 [file NA-003-D1NA00207D-s001.pdf]

Electronic Supplementary Material (ESI) for Nanoscale Advances.
This journal is © The Royal Society of Chemistry 2021

Electronic Supporting Information (ESI)

for

Thermally-insulating vermiculite nanosheets-epoxy nanocomposite paint as fire-resistant wood coating

Abimannan Sethurajaperumal[a‖], Anagha Manohar[a‖], Arghya Banerjee[a], Eswaraiah Varrla[a*], Hao Wang[b], and Kostya (Ken) Ostrikov[c*]

[a]*Nanosheets and Nanocomposites Laboratory, Department of Physics and Nanotechnology, SRM Institute of Science and Technology, Kattankulathur, Chengalpattu, Tamil Nadu, 603203, India.*

[b]*Centre for Future Materials, University of Southern Queensland, Toowoomba, QLD 4350, Australia.*

[c]*School of Chemistry and Physics and QUT Centre for Materials Science, Queensland University of Technology (QUT), Brisbane, QLD 4000, Australia.*

[‖]These authors are equally contributed to the work.

Methods

As described in the methods section, we have used the low-power bath sonication (70 W) to disperse the vermiculite nanosheets (ex-VN) in epoxy monomer and to disperse the hardener in ex-V/epoxy monomer. The ice bath conditions are always maintained to slow down the curing process particularly when the hardener agent is added. After the bath sonication, we immediately coated the formulated paint on the wood. As per the manufacturer's instructions, 3:1 ratio of epoxy and hardener should be mixed and kept idle for 24 hrs for curing to take place. In the first step of preparation, there is no chance for curing to takes place without hardener and in the second step, it is unavoidable to use homogenizer (mechanical, sonic etc.) to disperse the fillers and hardener throughout the epoxy and sonication induced curing is insignificant in such short intervals since we did not notice any changes in flow behavior of formulated paint which is highly likely if curing takes place. In our work, we have not coated any wood samples with bare ex-VN. To the best our knowledge, if we coat ex-VN on the wood directly, it may result in poor adhesion and the fire retardant properties may not get improved at all.

Phyllosilicates

Phyllosilicate minerals possess a variety of unique properties in terms of structure, adsorption characteristics, thermal and mechanical properties, and above all, being eco-friendly. It is composed of crystalline layers stacked upon one another and can be broken down into nanosheets. Among phyllosilicates, vermiculite is a promising material owing to its exceptionally low thermal conductivity of 0.129 W/mK which further reduces to 0.096 W/mK and structural stability at extremely high temperatures. It has a 2:1 layered structure composed of an octahedral layer consisting of magnesium ions sandwiched between two tetrahedral silica layers in which aluminium partially replaces silicon. The interlamellar space also contains water molecules and

other feebly bonded and exchangeable ions such as Na^+, K^+, Mg^{2+}, and Ca^{2+} which compensate for the charge deficiency due to replacement of silicon atoms with Al^{3+} ions.

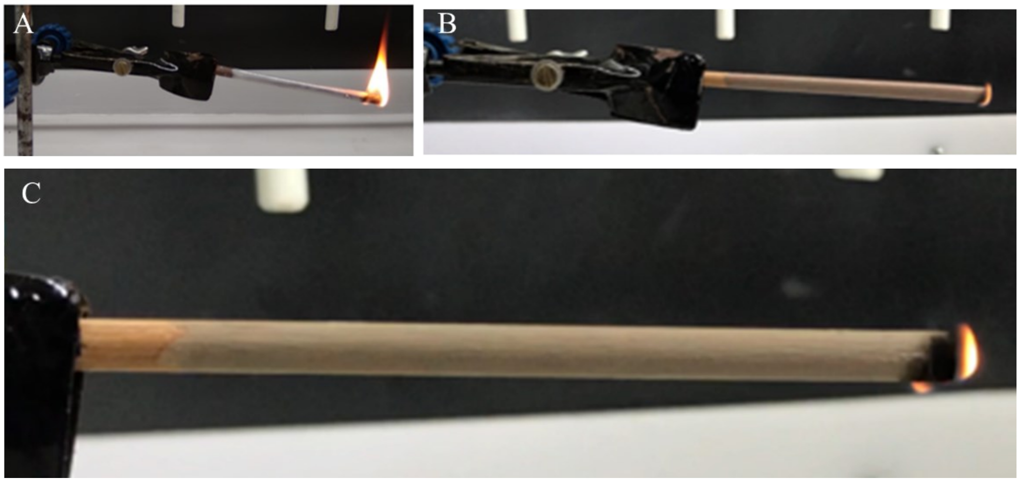

Fig. S1. Photographs of **A**. 5 mm rod wooden samples with bare epoxy coated, 5 times coated (~80 μm), **B**. 10 wt.% ex-VN/epoxy (5 times coated) and **C**. 30 wt.% ex-VN/epoxy (5 times coated) after performing fire test for 6 s.

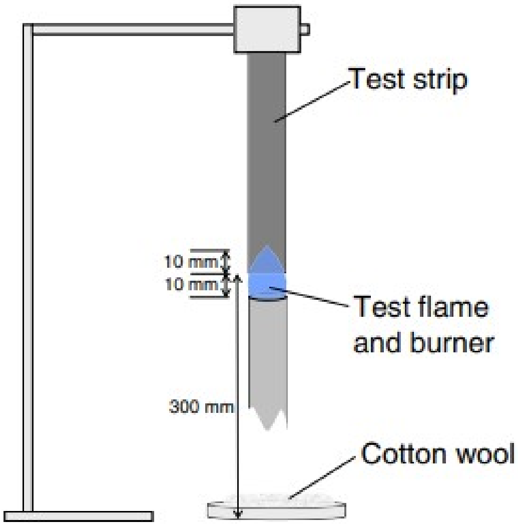

Fig. S2. Schematic of UL 94 Test set up

The sample is placed vertically and is exposed to flame from lighter or burner wherein the sample is subjected to a small heat flux as opposed to the cone calorimetry wherein it can go up to 50 kW/m^2. The flame is applied to the bottom free end of the sample and the flame is 10 mm away from the sample and this distance is maintained throughout the experiment. An absorbent cotton tissue is kept beneath the samples to check for any material dripping during combustion. The minimum sample size is around 10 mm in this standard test. The schematic of experimental set up is shown in Figure S2.

CRITERIA FOR CLASSIFICATION	V_0(s)	V_1(s)	V_2 (s)
After flame time (T_1 or T_2)	≤10	≤30	≤30
Total after flame time (T1 +T2)	≤50	≤250	≤250
After glow time after T2	≤30	≤60	≤60
After glow of any specimen upto clamp	NO	NO	NO
Cotton bud at the bottom ignited by flaming drips	NO	NO	YES

Table 1. Table showing the criteria for classification of fire-resistant materials based on flame extinguishing time and dripping.

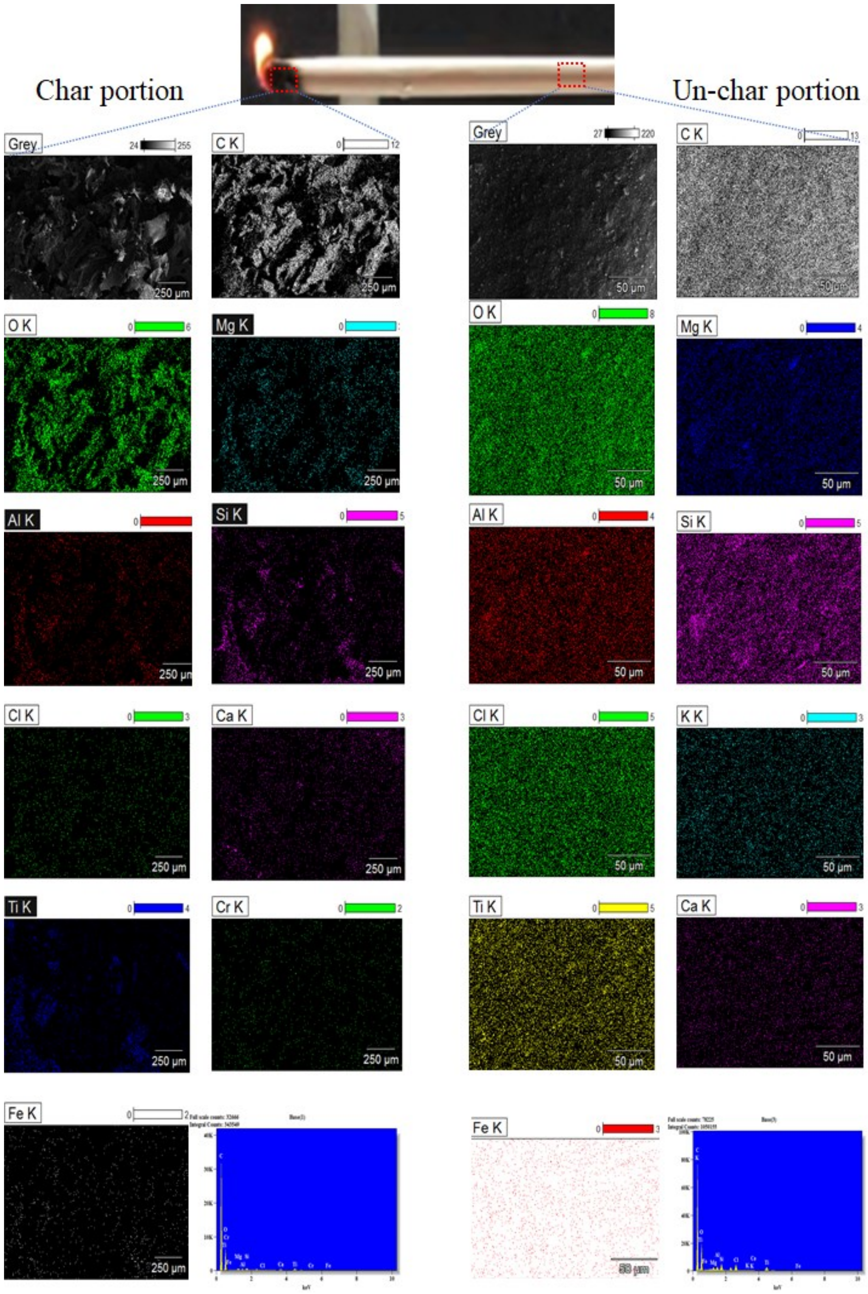

Fig. S3. Elemental mapping of 20 wt. % ex-VN filled epoxy coated wooden rod's char portion and its EDAX spectrum and un-charred region.

The next parameter to be discussed here is the flame height and behaviour. In case of the controlled sample and the bare wood sample we observe that as the combustion progresses through the length of the wood the flame height and the brightness of the flame keeps increasing and burns more

vigorously, spreading through the entire rod accompanied by a lot of smoke release with the passage of time. However, for the ex-VN filled epoxy no vigorous burning behavior was observed.

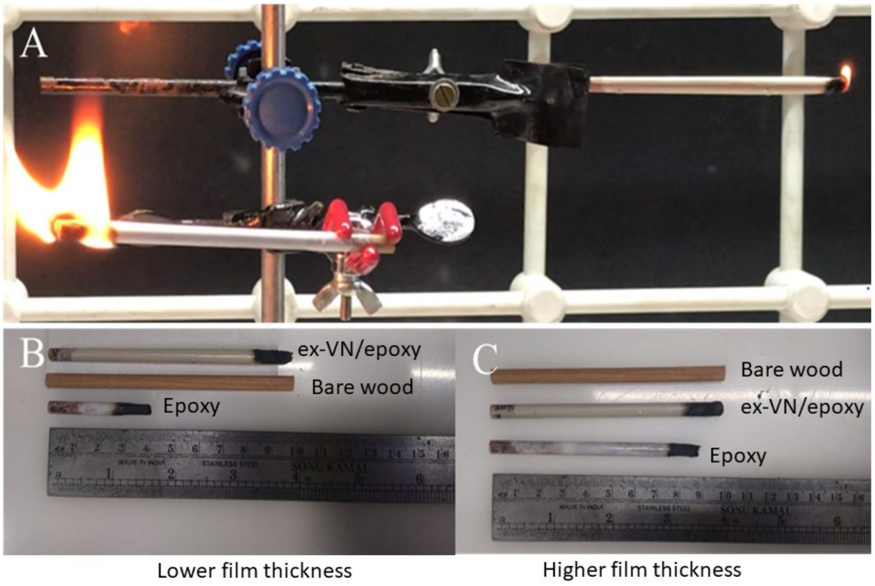

Fig. S4. A. Photograph showing greater flame spread on controlled (epoxy alone) sample (right) compared with 20 wt. % ex-VN/epoxy coated wood (left) after igniting at the same instant. **B & C** Photograph taken post combustion showing the length of sample burnt due to propagating flame for 5 (B) and 7 (C) times coated with 20 wt. % ex.VN/epoxy sample along with controlled epoxy and bare wood sample.

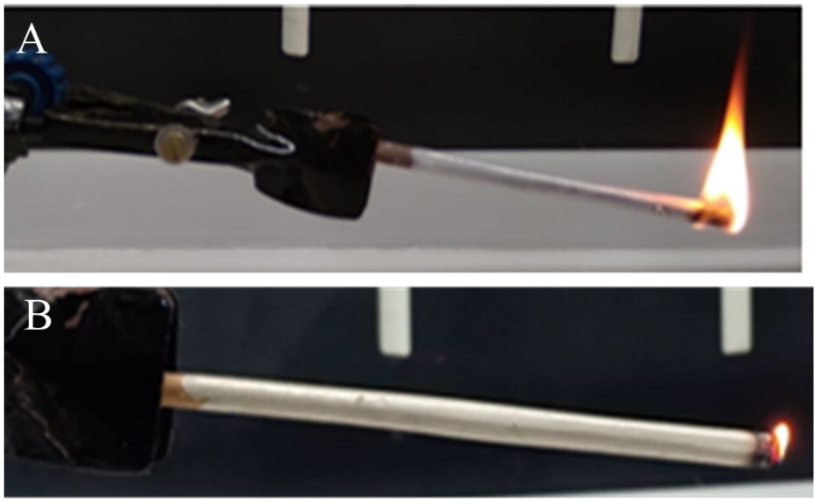

Fig. S5. Photograph showing flame height of **A**. Controlled sample (wood with epoxy without ex-VN) and **B**. 20 wt.% ex-VN/epoxy coated wooden rod after igniting it for the same fixed time.

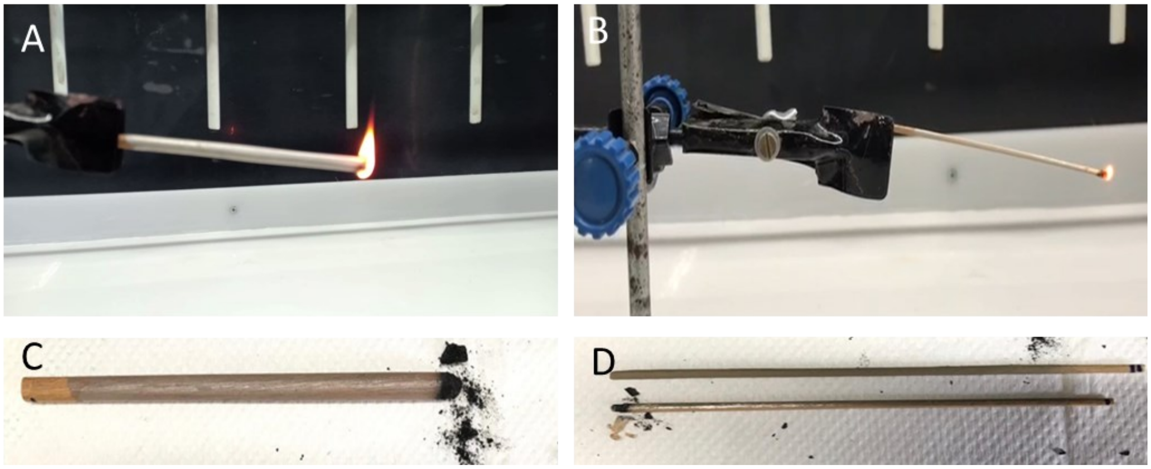

Fig. S6. A. Photograph showing bright flame for 5 mm sample with 20 wt.% ex-VN/epoxy coated wood, **B**. Photograph showing dimmed flame for 3 mm sample with 20 wt.% ex-VN/epoxy coated wood **C.** Photograph taken post combustion showing higher mass degradation for 5 mm rod sample. **D.** Photograph taken post combustion showing minimal mass degradation for 3 mm rod sample.

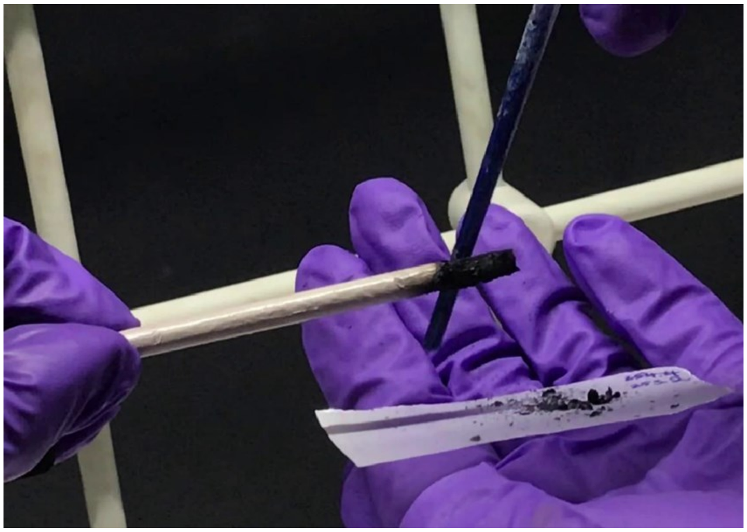

Fig. S7. Photograph of wooden rod after combustion test displaying loose charred powder.

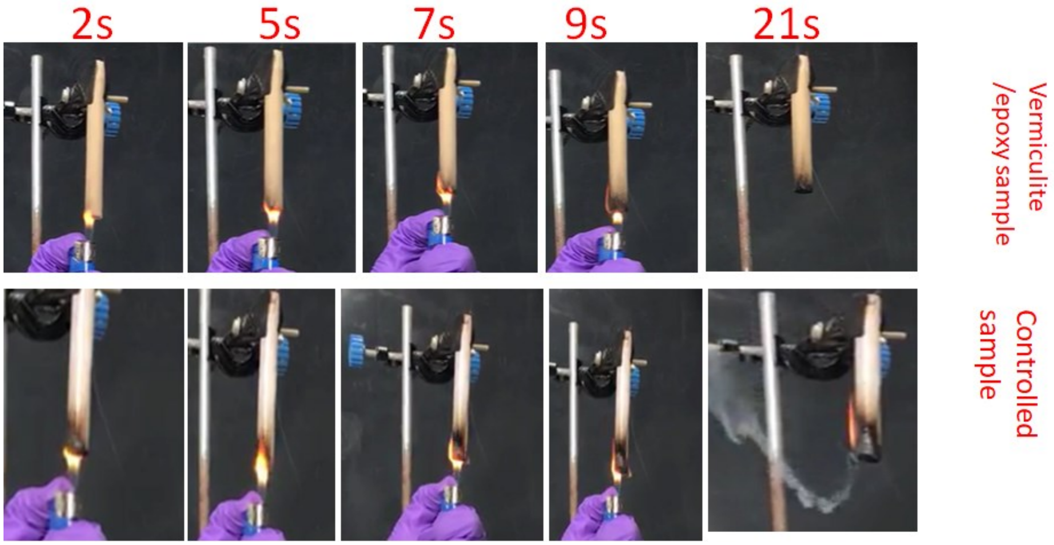

Fig. S8. Photographs of ex-VN/epoxy coated wooden rods (10 cm x 10 mm) after first ignition in UL-94 test.

Flame extinguish time will not increase by adding more ex-VN but the observed variation within the 10-30 wt.% is due to dispersion of nanofiller in viscous epoxy. As we increase the percentage of ex-VN, the flame extinguish time of 10, 20 and 30 wt.% ex-VN/epoxy composite is decreased as shown in Figure 6A (grey colored) when compared with controlled sample (0 wt.% ex-VN in epoxy). The observed variation in flame extinguish time of different weight percentages of ex-VN/epoxy composites is due to several reasons. In polymer composites, as the mass of nanofiller is increased, percolation is achieved at a certain weight percentage of nanofiller and above that threshold, the composite remains in transition state with improved properties.

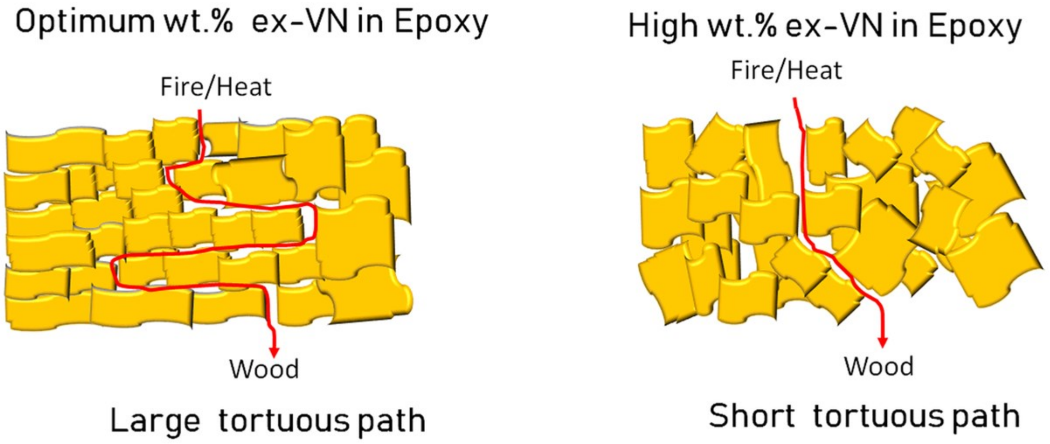

Fig. S9. Schematic of fire/heat tortuous path at low and high weight percentages of ex-VN in epoxy composites.

However, the dispersion, orientation and aggregation of nanofillers is completely random and it depends on the process of preparing the nanocomposites and this will show variation in fire-retardant properties of polymer composites. This could be improved if the exfoliated vermiculite nanosheets are functionalized with molecules which are compatible with epoxy monomers and then polymerizing it. However, this work requires more time with greater focus on understanding the interface between epoxy and nanofiller.

The afterglow time shows a more or less consistent behavior. Afterglow is the slow burning of material/composite even after the ignition source is removed. Afterglow time is suppressed upon increasing addition of fire retardant nanofiller in the composite matrix as we have noticed. Exfoliated vermiculite nanosheets might serve as a condensed phase flame retardant and the condensed phase can alter the pyrolysis process. That's why, on increasing nanofiller addition we observed a reduction in afterglow time but variation in afterglow time within ex-VN/epoxy samples could be the same reason as explained above [1]. Exfoliated vermiculite nanosheets prevents the formation of a flammable air-fuel mixture via condensed-phase reaction [2].

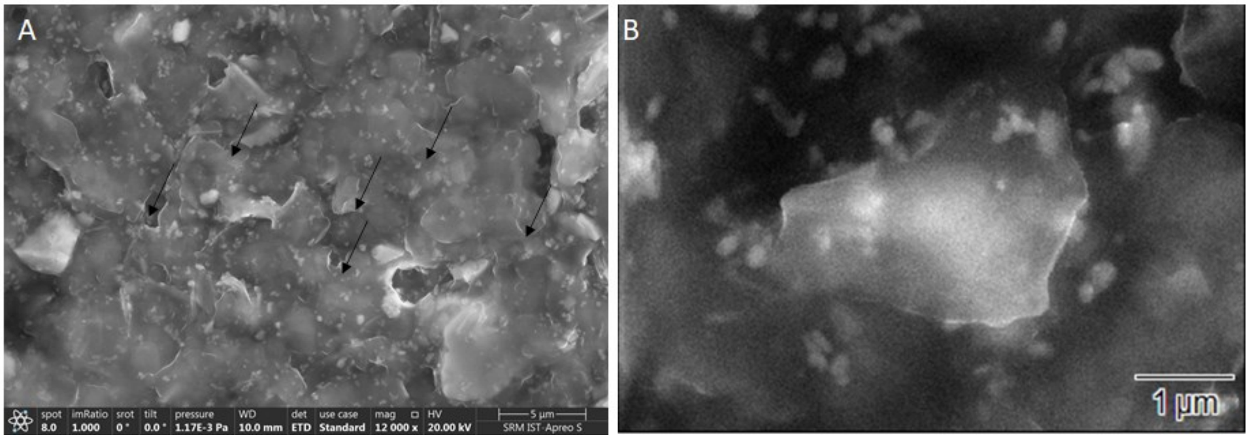

Fig. S10. A-B) Low-resolution and High-resolution field emission scanning electron micrograph of the ex-VN (20 wt.%) filled epoxy nanocomposite.

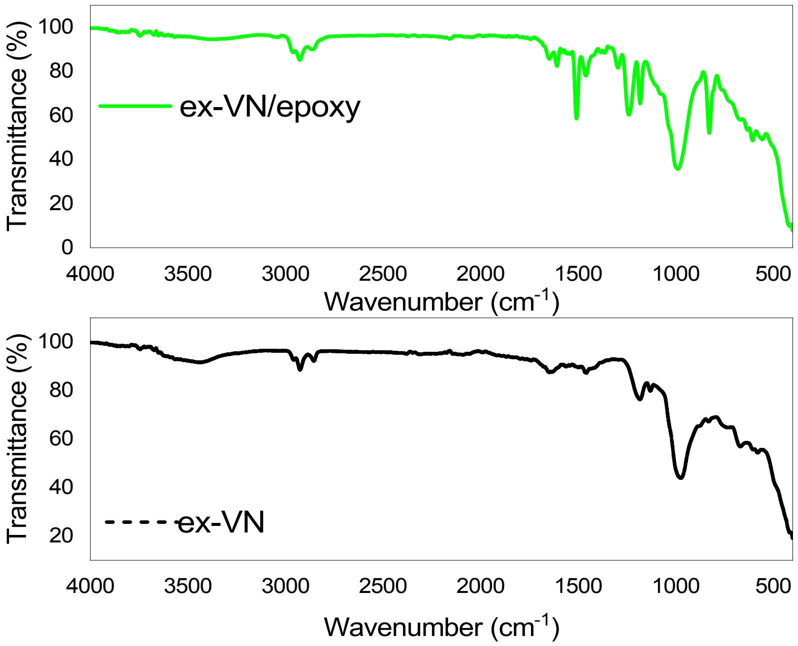

Fig. S11. FTIR Spectrum (ATR mode) of exfoliated vermiculite nanosheets and its epoxy nanocomposite.

Vermiculite has tetrahedral silicate atoms sandwiched by two octahedral Mg atoms. As shown in the FTIR spectrum, exfoliated vermiculite nanosheets and its epoxy composite has peaks in lower wavenumber 1000-400 cm^{-1}, confirming the presence of metals (Mg, Si) and peak position at ~830 cm^{-1} due to the octahedral silicate layer trivalent central atoms bending vibrations. However, we observed little shift in lower wavenumber ~827 cm^{-1} for ex-VN/epoxy nanocomposite which might be due to the epoxide functional groups non-covalently interacting with the atoms. Other modes of octahedral silicate atoms vibration confirmed by 1460 cm^{-1}, 669 cm^{-1}, 605 cm^{-1} peaks due to the Si-O-Si asymmetric stretching vibrations, deformation and bending modes of Si-O atoms, coordination bending between tetrahedral Si-O into octahedral O-Mg bonds respectively. Here we have used naturally occurring vermiculite for the exfoliation process. There is a possibility that it contains a minor amount of hydrocarbon. Peaks at 2863, 2927, and 2961 cm^{-1} are attributed to the presence of hydrocarbons in vermiculite. Presence of water molecules in vermiculite and ex-

VN/epoxy composite confirmed by H_2O molecule bending mode vibrations at ~ 1647 cm^{-1}. The presence of epoxy in vermiculite-epoxy nanocomposites confirmed by transmittance peaks at 1508 cm^{-1} and 1608 cm^{-1} attributed to stretching vibration of C-C and C=C aromatic rings. Exfoliated vermiculite nanosheets peaks' positions match with ex-VN/epoxy nanocomposite peak position. This suggests that ex-VN is non-covalently bonded with the epoxy matrix. However, bare this has one strong peak observed at 975 cm^{-1} arising due to the silicate atoms stretching vibrations and this peak shift towards higher wavenumber (990 cm^{-1}) in nano-composites might be due to the creation of amorphous silicon with three-dimensional network formed [3, 4]

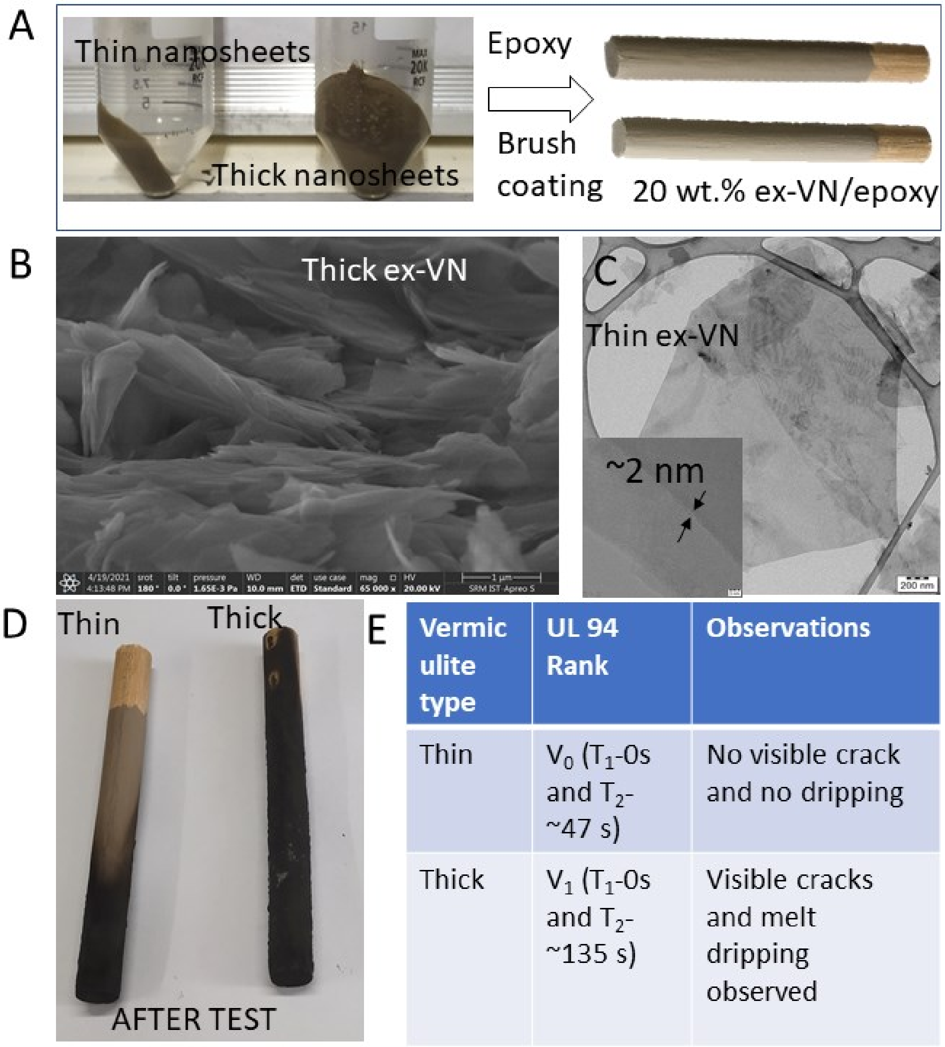

Vermiculite type	UL 94 Rank	Observations
Thin	V_0 (T_1-0s and T_2-~47 s)	No visible crack and no dripping
Thick	V_1 (T_1-0s and T_2-~135 s)	Visible cracks and melt dripping observed

Fig. S12. A) Photograph of the thin and thick exfoliated vermiculite nanosheets (left) and the ex-VN/epoxy coated wood samples (right), B) SEM image of thick ex-VN, and C) TEM image of thin ex-VN (inset: representative TEM image of the sheet at the edge), D) Photograph of the 20 wt.% ex-VN/epoxy coated (thick and thin) wood samples after UL-94 test and E) Rank and observations from UL-94 test on coated samples.

We have experimented to see the effect of ex-VN's thickness on fire-retardant property. For this, bulk vermiculite is exfoliated in a water-surfactant system, and the samples are separated as thick (sediment) and thin (supernatant). SEM and TEM characterize these two sets of samples to measure the thickness, and we found that thin nanosheets are 2-4 nm (from TEM image) and thick nanosheets are ~50 nm (from SEM image). These two fillers (20 wt.%) are incorporated in epoxy and coated on wood in the same way and the UL-94 test is performed, and the observations are kept in **Figure S12.** We found that thin ex-VN/epoxy coated samples could extinguish the flame immediately in the first ignition and less than ~50 s in the second ignition, thus classified as V_o class.

In contrast, thicker nanosheets could take 135s in the second ignition, thus classified as V_1 class. The reason could be thicker nanosheets may not provide sufficient enough tortuous path as the network is limited. In contrast, thinner nanosheets have a high surface area, and the number of layers is relatively high, making it more resistant to the propagation of heat.

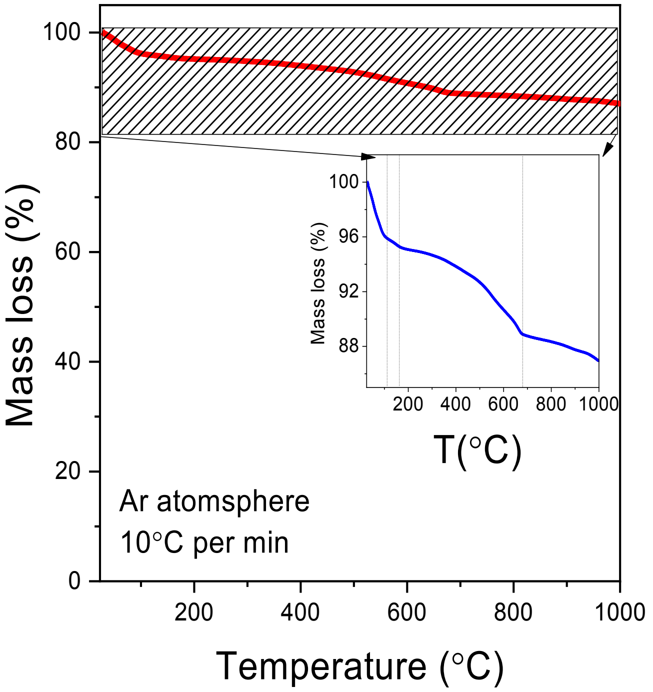

Fig. S13. TGA of exfoliated vermiculite nanosheets in Argon atmosphere from room temperature to 1000°C at the rate of 10°C/min (STA 7000, HITACHI).

As shown in the result, the exfoliated vermiculite nanosheets are highly stable at high temperatures and there is ~13% mass loss in this measured temperature range in different stages. The mass loss of ~3.9% between RT to 100°C corresponds to the removal of volatile compounds and water molecules adsorbed on the surface. From 100°C to 169°C, the mass loss of ~1 % is due to the decomposition of hydrate molecules and the mass loss of ~6.3% when the temperature is changed from 169°C to 674°C is due to the removal of intercalated cations in vermiculite. The final stage (674 to 1000 °C) mass loss is ~ 1.3%. Reduction of this mass might be a result of the thermal decomposition of very few oxides into other by-products.

REFERENCES

1. Giri, R., L. Nayak, and M. Rahaman, *Flame and fire retardancy of polymer-based composites.* Materials Research Innovations, 2021. **25**(2): p. 104-132.
2. Muiambo, H.F., W.W. Focke, and J.K.O. Asante, *Flame retardant properties of polymer composites of urea complex of magnesium and vermiculite.* AIP Conference Proceedings, 2019. **2055**(1): p. 050011.
3. Omar, M.F., et al., *Thermal properties of polypropylene/muscovite layered silicate composites: effects of organic modifications and compatibilisers.* Journal of Composite Materials, 2014. **49**(10): p. 1195-1209.
4. Gu, S., et al., *Selective Adsorption of Pb(II) from Aqueous Solution by Triethylenetetramine-Grafted Polyacrylamide/Vermiculite.* Materials, 2018. **11**(4): p. 514.
